# The Association between Sleep Quality and Depressive Symptoms among Stroke Survivors and Caregivers

**DOI:** 10.3390/healthcare12010058

**Published:** 2023-12-26

**Authors:** Lisa A. Babkair, Hanadi Huri, Walaa Alharbi, Yara Turkistani, Ruba Alaslani, Nisreen Alandijani, Fatimah Hamad Hakami

**Affiliations:** 1 Faculty of Nursing, King AbdulAziz University, Jeddah 21589, Saudi Arabia; halihuri@stu.kau.edu.sa (H.H.); wmalharbi0001@stu.kau.edu.sa (W.A.); yabbasturkistani@stu.kau.edu.sa (Y.T.); ralaslani0025@stu.kau.edu.sa (R.A.); 2 King Abdullah Medical Complex, Jeddah 23816, Saudi Arabia; nalandijani@moh.gov.sa; 3 King Fahad General Hospital, Jeddah 23325, Saudi Arabia; fatimahhh@moh.gov.sa

**Keywords:** sleep quality, depressive symptoms, stroke survivors, caregivers

## Abstract

Background: Stroke is a serious health problem that can lead to the development of depressive symptoms, sleep disturbances, and functional dependence in stroke survivors. The change in stroke survivors’ quality of life results in negative health outcomes for stroke survivors and caregivers. This study aims to investigate the association between sleep quality and depressive symptoms among stroke survivors and caregivers in Saudi Arabia. Methods: A cross-sectional design was used to collect data from 100 stroke survivors and 80 caregivers using the patient health questionnaire and Pittsburgh Sleep Quality Index. Results: 43% of the stroke survivors reported depressive symptoms and 65% had poor sleep quality; 21% of the caregivers reported depressive symptoms and 46% reported poor sleep quality. A strong association between sleep quality and depressive symptoms was detected for the stroke survivors and caregivers: (*r* = 0.885, *p* < 0.001); (*r* = 0.669, *p* < 0.001), respectively. A strong association was found between caregivers’ depressive symptoms and patient post-stroke depressive symptoms (r = 0.502, *p* < 0.001). A moderate association was found between stroke survivors’ sleep quality and caregivers’ sleep quality (*r* = 0.407, *p* < 0.001). There was a moderate association between stroke survivors’ depressive symptoms and caregivers’ sleep quality (*r* = 0.456, *p* < 0.001). Moreover, there was a moderate association between stroke survivors’ sleep quality and caregivers’ depressive symptoms (*r* = 0.492, *p* < 0.001). Conclusion: Poor sleep quality and depressive symptoms are common among stroke survivors and caregivers. This study found an association between the two variables. Therefore, depressive symptoms and sleep disturbances in stroke survivors and caregivers should receive more attention. Future research should focus on an interventional study to enhance sleep quality and decrease the risk of depressive symptoms.

## 1. Introduction

Stroke is the second leading cause of death. Each year, 15 million people worldwide suffer a stroke, of which one-third of them die and another one-third are left with long-term disability, while the remainder recover and restore normal function [1]. Stroke is a devastating disease, which places a physical and emotional burden on stroke survivors and caregivers. Stroke survivors experience physical disability, functional dependence, and stroke-related emotional and sleep disorders [2]. The emotional health of stroke survivors is as important as their physical health. Post-stroke depressive symptoms (PSDS) are a common phenomenon among stroke survivors. They affect approximately one-third of stroke survivors at any one time after stroke [3].

In Saudi Arabia, the incidence of stroke is drastically increasing. A recent study predicts an increase in first strokes in Saudi Arabia over a 10-year period within the range of 57–67% [4]. The annual incidence of stroke in Saudi Arabia is 29 stroke cases for every 100,000 people [5]. The aging population raises concerns for future growth in the number of strokes that require specialized stroke care. Strokes lead to a high burden due to an increasing incidence rate with a mortality rate that is projected to be nearly double by 2030 [6]. As a result, a major challenge in the stroke healthcare system is anticipated in Saudi Arabia. Currently, there are over 350 hospitals with a limited number of stroke centers in Saudi Arabia [7]. In fact, only approximately 5% of stroke patients in Saudi Arabia are admitted to acute stroke units and receive advanced stroke care [8]. However, the Ministry of Health is currently activating the track of the comprehensive stroke treatment program using the latest internationally available medical technologies such as Telestroke that could help in early stroke management.

Several studies reported a number of post-stroke complications, such as sleep disturbance, functional dependence, PSDS, and physical disability [9,10]. Sleep is an important physiological function in every human being, especially for patients requiring the appropriate healthcare after being discharged due to acute illness. Many stroke survivors experience sleeping difficulties, such as insomnia, sleep apnea, and a long daytime sleep duration, which affects their quality of life [10]. According to previous studies, there is a wide prevalence of post-stroke sleep disturbance, ranging from 25% to 85% [10,11,12,13]. Moreover, sleep quality has a direct impact on people’s health and can worsen their clinical symptoms [13]. Poor sleep quality can result in negative effects on stroke survivors’ functional dependency, which can cause delays in stroke patients’ recovery [10,13].

In Saudi Arabia, the prevalence of PSDS ranges from 17 to 63.3%, indicating that depression after stroke is a common emotional disorder [14]. However, PSDS is still unrecognized among stroke survivors. Depression after stroke affects patients’ rehabilitation and delays recovery if it is not discovered and treated early. Patients who develop PSDS experience impairments in physical activity, delays in cognitive recovery, a decline in their quality of life [10], and increased mortality [3]. Furthermore, research has found that PSDS is more common in people with severe disabilities [15]. Additionally, there are statistically significant positive connections between depression symptoms and poor sleep quality *r* = 0.553, *p* < 0.01 and anxiety *r* = 0.517, *p* < 0.01 [16].

Post-stroke depression is the common term used to classify mood disturbances among patients who had strokes. Depression after stroke is classified as a mood disorder resulting from a general medical condition with depressive features, major depressive episodes, or mixed-mood features [17].

Previous studies also confirmed a relationship between social support and PSDS. Social support is known as a multidimensional construct. It has been classified as perceived social support and received social support. It is also classified based on its types including information, instrumental, financial, and emotional support, and can be provided by family, friends, colleagues, and medical staff. In this study, we define social support as the provision of material support and emotional support by caregivers to improve stroke survivors’ ability to manage stressful events [16]. Stroke survivors are discharged from the hospital and usually supported by their family members, who provide direct care and emotional support. Caregivers face multiple challenges while providing care for stroke survivors with functional dependency [18]. Multiple studies have confirmed that caregivers might suffer from depression and other psychological problems. A prospective longitudinal study reported that stroke survivors’ caregivers have a high risk of developing depressive symptoms over time [19]. Therefore, caregivers are also in need of support to help them cope with post-stroke stressors and sudden life changes.

According to a previous study, all family caregivers deal with psychological problems such as stress and depression. The study also discovered that caregivers face a variety of difficulties, such as sleep deprivation, financial difficulties, a lack of a support structure, intense caring, and over-dependence [20]. Another research study revealed a substantial relationship between caregivers’ poor sleep quality and an increased burden (*p* = 0.004) and psychological distress (*p* = 0.004) [21]. Furthermore, another study revealed that the sleep duration, satisfaction, and sleep quality of informal caregivers for stroke survivors declined by 30% after starting to provide care [22].

Caregivers often feel unprepared for their new role, and this may cause high levels of anxiety and depression, which have a negative impact on the caregiver’s health and wellbeing. The health of caregivers and the support they receive play an important role in stroke survivors’ recovery. However, family health disturbances due to sudden changes in the condition of stroke survivors might result in negative health outcomes.

PSDS is a serious emotional disorder among stroke survivors and affects patient health outcomes negatively. Several factors are associated with depressive symptoms such as lack of social support from family and friends, poor sleep quality, and physical disability. Caregivers are also at great risk of developing depressive symptoms and sleep disturbance because of sudden changes in the life of a loved one. Therefore, caregivers also need special care and attention to be screened for depressive symptoms for early identification. Limited studies have investigated the association between sleep quality and PSDS among stroke survivors and their caregivers in Saudi Arabia. The results of this study offered knowledge and recommendations for further investigation to enhance the follow-up treatment plan, early screening for post-stroke depression and sleep quality, and positive health outcomes.

This study was conducted to answer the following research questions: What is the type of association between PSDS and poor sleep quality among stroke survivors and caregivers? The hypotheses of this study were (1) an association between PSDS and poor sleep quality among stroke survivors, (2) an association between depressive symptoms and poor sleep quality among caregivers, (3) an association between PSDS among stroke survivors and caregivers’ depressive symptoms, (4) an association between stroke survivors and caregivers’ poor quality of sleep, (5) an association between PSDS among stroke survivors and caregivers’ poor quality of sleep, and (6) an association between stroke survivors’ poor quality of sleep and caregivers’ depressive symptoms.

The aim of this study is to investigate the association between sleep quality and depressive symptoms among stroke survivors and caregivers.

## 2. Materials and Methods

### 2.1. Study Design and Participants

A quantitative, descriptive, cross-sectional design was used to investigate the association between sleep quality and depressive symptoms among stroke survivors and caregivers. This study was conducted at King Fahad General Hospital (KFGH) in Jeddah and King Abdullah Medical Complex (KAMC) in Jeddah. KFGH is considered one of the biggest MOH hospitals in the western region of Saudi Arabia, with a bed capacity of 628. King Abdullah KAMCJ is a 500-bed hospital. The reason for selecting these hospitals is because both hospitals are eligible to admit stroke patients and provide stroke care.

A convenience sample of stroke survivors and caregivers was selected from the two hospitals. This study included stroke survivors who had a stroke more than one month earlier to ensure that survivors are medically stable and prevent the acute stroke stage, which might affect the depression onset. Also, survivors who were able to sufficiently comprehend and communicate in Arabic were included. Individuals with conditions such as cognitive impairment, dementia, and aphasia that would limit their ability to complete a survey were excluded. Caregivers who were ≥18 years old, living with stroke survivors, and provided home healthcare were eligible to participate in the study.

### 2.2. Measurement Tools

#### 2.2.1. Sociodemographic Questionnaire

During quantitative data collection, participants were asked to complete a brief sociodemographic questionnaire. The sociodemographic form includes variables that may influence sleep quality and PSD such as age, gender, marital status, living status, literacy level, medical history, stroke duration, and monthly income. Caregivers completed the same sociodemographic questionnaire, with some extra questions related to caregivers such as their relation to the patient.

#### 2.2.2. Patient Health Questionnaire (PHQ-9)

The PHQ-9 is a brief tool used to measure PSDS. The PHQ is a self-administered tool and takes approximately 2–5 min to complete [23]. The American Heart Association/American Stroke Association (AHA/ASA) recommends using a PHQ-9 questionnaire to screen patients for PSDS because of the instrument’s brevity in comparison to other depression scales, such as the Beck Depression Inventory [3]. The PHQ-9 is a reliable and valid instrument for measuring PSDS; Cronbach’s alpha coefficient is 0.839 [24,25]. This study used the Arabic version, and PSDS was measured as continuous data using a total PHQ-9 score, while the presence or absence of PSDS was treated as binary data at a cutoff point of ≥10.

#### 2.2.3. Pittsburgh Sleep Quality Index (PSQI)

This is a survey that rates and evaluates sleep quality and disturbances over one month. PSQI is a self-reported questionnaire and contains nineteen different variables that generate seven components: subjective sleep quality, sleep latency, sleep length, sleep disruptions, habitual sleep efficiency, usage of sleeping medications, and daytime dysfunction [26]. The survey has 24 questions, 19 of which are self-reporting, and 5 of which, if any are available, are evaluated by a sleeping partner but are not considered when determining the results. Four items demand a numerical response, but the majority of the items are scored on a 4-point scale [27]. The total score was calculated by summing the results for the seven components; it ranged from 0 to 20. The higher the result, the poorer the sleep quality [28]. According to psychometric analyses, the PSQI’s reliability and validity have been confirmed by multiple studies, and it can be used to accurately and consistently measure sleep disorders in clinical populations of Arabic speakers [28]. Cronbach’s alpha coefficient is 0.74, indicating good internal consistency of the scale [28]. The Arabic version of the PSQI was used in this study after obtaining permission from the author. The Arabic version is also reliable and valid for use among stroke survivors and caregivers. The poor sleep quality was treated as binary data at a cutoff point of >5 [26].

### 2.3. Data Collection Procedures

Ethical approval was obtained from the Faculty of Nursing at King Abdulaziz University and the Ministry of Health for the previously mentioned hospitals. The researchers identified stroke survivors who were eligible to participate in this study. Next, an effort was made by the researchers by contacting the participants and explaining the study to them over a phone call. Participants who agreed to participate in the study and met the eligibility criteria were asked to sign a consent form via an electronic survey link. After that, a link to SurveyMonkey that included three questionnaires was sent to the participants. Participants who faced difficulties in completing an online survey using their smartphone were offered the option of allowing the researchers to complete the survey on their behalf by reading questionnaire items over the phone. Researchers wrote notes while completing the survey over the phone in case of data collection interruption due to technical issues.

### 2.4. Statistical Analysis

To determine the adequate sample size for the study, an a priori power analysis was conducted using G*Power version 3.1 to determine the minimum sample size required to investigate the study research questions. The results indicated that the required sample size to achieve 80% power for detecting a medium effect, at a significance criterion of α < 0.05, was N = 80 for Pearson’s correlation [29]. Descriptive statistics were used to explain the sample and the instruments used in this study. Means, standard deviations, and frequency were used to analyze the demographic variables. Means, standard deviations, medians, and ranges were used for PHQ-9 and PSQI. Pearson correlations were used to test bivariate associations between PHQ scores and PSQI.

## 3. Results

A sample of 100 patients and 80 caregivers was enrolled from King Fahad General Hospital (KFGH) and King Abdullah Medical Complex (KAMC). The sample included patients who were diagnosed with stroke more than one month prior. Table 1 shows the sociodemographic properties of the sample in this study. The mean age of patients and caregivers is 57.32 years, *SD* = 14.282 and 40.43 years, *SD* = 12.152, respectively. The age range was between 24 and 85 years for patients and from 17 to 76 years for caregivers.

The majority of the patients were Saudi (89%), male (59%), and married (66%). The majority of the patient sample had a high school level of education (35%) and most of the sample were housewives (32%). The average stroke onset was 21 months ago, and approximately 44% of the patients had a stroke more than one year prior to the study.

The majority of the caregivers were Saudi (86.3%), female (56.3%), and married (61.3%). The majority of caregivers had a university degree (57.5%), and more than half of the sample were employed (57.5%). The average number of people living in the caregiver’s household was 4.21, and the average length for which they provided care for the stroke patient was 23 months. Most of the caregivers provided post-stroke care for their mother (36.3%), father (33.8%), spouse (15%), or sibling (8.8%).

Table 2 shows the medical conditions of the stroke survivor sample. The most common comorbidities among stroke survivors were hypertension (65%), diabetes (60%), hyperlipidemia (35%), previous stroke (18%), and smoking and cardiovascular disorders (16%). Regarding the caregivers, the most common medical conditions were hypertension (25%), smoking (18.8%), diabetes (16.3%), and hyperlipidemia (11.3%).

Table 3 shows the PHQ-9 score for stroke survivors and caregivers. More than half of the patient sample had moderate depressive symptoms (30%) and mild depressive symptoms (23%), whereas approximately 35% of the caregivers had mild depressive symptoms and 16.3% had moderate depressive symptoms.

Table 4 shows that the average score for depressive symptoms for the patient sample is 9.56 (*SD* = 5.82) and ranges from 0 to 23, while the average score for depressive symptoms for the caregiver sample is 6.96 (*SD* = 4.753), ranging from 0 to 19. Furthermore, the average sleep quality score (PSQI) for the patient sample is 7.85 (*SD* = 3.622), ranging from 0 to 17. However, the average sleep quality score for the caregiver sample is 6.16 (*SD* = 3.046), ranging from 0 to 14.

Table 5 shows the prevalence of poor sleep quality and depressive symptoms. Among the patient sample, 43% had a PHQ-9 score ≥ 10 and 21.3% had a PHQ-9 score ≥ 10 in the caregivers’ sample, which indicates the presence of depressive symptoms. Furthermore, approximately 65% of the patient sample and approximately 46.3% of caregivers had poor sleep quality, with PSQI > 5.

**Figure 1:**
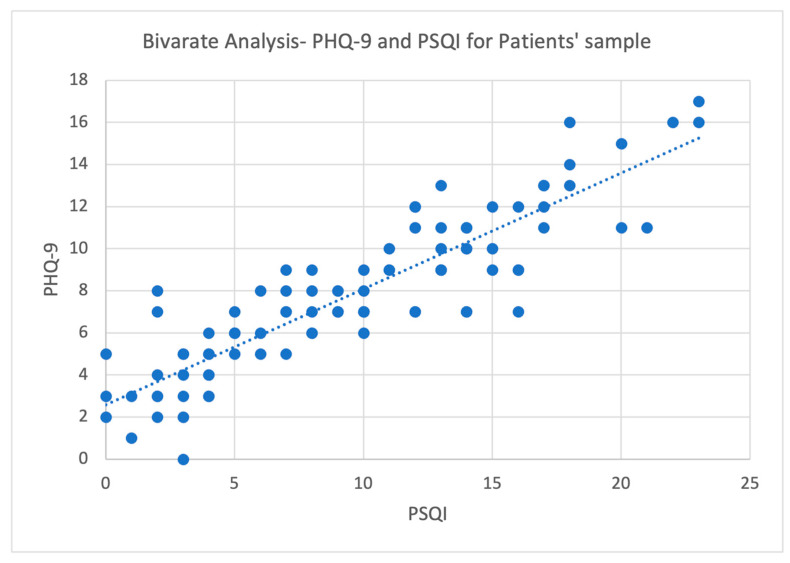
Pearson correlation of PSQI and PHQ-9 for patients.

**Figure 2:**
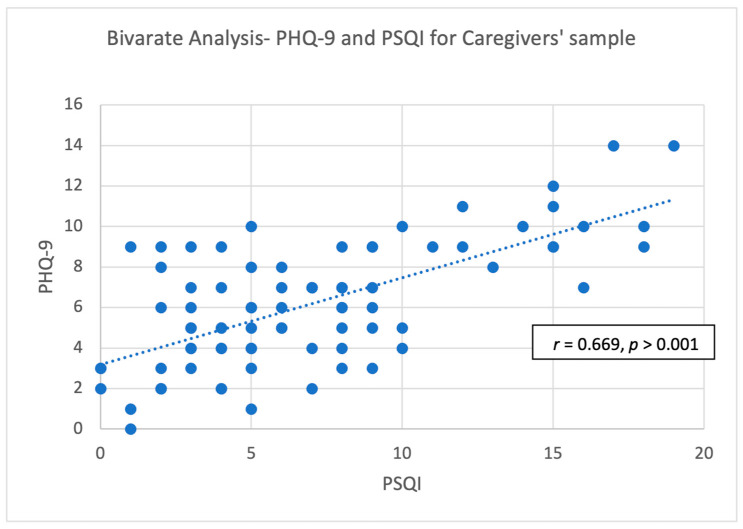
Pearson correlation of PSQI and PHQ-9 for caregivers.

Table 6 and Figure 1 and Figure 2 show a bivariate comparison between PSDS and sleep quality. There was a strong positive association between depressive symptoms and sleep quality for the patient sample (*r* = 0.885, *p* < 0.001) and caregivers (*r* = 0.669, *p* < 0.001). A strong association was found between caregivers’ depressive symptoms and patients’ post-stroke depressive symptoms (*r* = 0.502, *p* < 0.001). There was a moderate positive association between stroke survivors’ sleep quality and caregivers’ sleep quality (*r* = 0.407, *p* < 0.001). There was a moderate positive association between stroke survivors’ depressive symptoms and caregivers’ sleep quality (*r* = 0.456, *p* < 0.001). Moreover, there was a moderate positive association between stroke survivors’ sleep quality and caregivers’ depressive symptoms (*r* = 0.492, *p* < 0.001).

## 4. Discussion

The primary findings of this study were based on an analysis conducted on 100 stroke survivors and 80 caregivers, as follows: 43% of the patients had (PHQ-9) score ≥10, which indicates the presence of depressive symptoms, with an average score of 9.56 (*SD* = 5.82). In addition, 21% of the caregiver sample reported depressive symptoms with a (PHQ-9) score ≥10, with an average score of 6.96 (*SD* = 4.753). Furthermore, 65% of the patient sample reported poor sleep quality, with a (PSQI) score ≥5, with an average of 7.85 (*SD* = 3.622). Moreover, 46% of the caregiver sample reported poor sleep quality, with a (PSQI) score ≥5 and an average of 6.16 (*SD* = 3.406).

Another main result of this study is the strong positive association between depressive symptoms and sleep quality for the patient sample (*r* = 0.885, *p* < 0.001) and for caregivers (*r* = 0.669, *p* < 0.001). Furthermore, there was a strong association between caregivers’ depressive symptoms and patients’ post-stroke depressive symptoms (*r* = 0.502, *p* < 0.001). There was a moderate positive association between stroke survivors’ sleep quality and caregivers’ sleep quality (*r* = 0.407, *p* < 0.001). There was a moderate positive association between stroke survivors’ depressive symptoms and caregivers’ sleep quality (*r* = 0.492, *p* < 0.001). Depression after stroke is a common phenomenon among stroke survivors. Most of the survivors suffer from emotional disturbance related to poor stroke outcomes such as physical independence.

The prevalence of PSDS in the study was 43%. This finding was higher compared to the results of previous studies. A cross-study reported that the pooled prevalence of depressive symptoms was 35% [15]. Another cohort study reported that the prevalence of depressive symptoms after a stroke was 27.5% [11]. A cross-sectional study obtained a similar result of PSDS of 45% [9] and another study obtained a slightly higher result of PSDS of 46.2% [16]. Some previous studies measured post-stroke depression at three months and others used either large or small sample sizes, which might explain the discrepancy between our findings and the literature [11,15,16]. One study enrolled 408 stroke survivors and followed them for 12 months at different measurement points [10]. Another study enrolled stroke survivors from an in-patient rehabilitation center, which might add to the discrepancy with our findings [16]. Another study focused on stroke survivors who were aged >55 years old and were ambulatory in the community [12]. Furthermore, pre-existing depression was not assessed in this study, which might add some limitations to the study findings.

As for the caregiver sample, 21% reported moderate depressive symptoms. This finding was lower compared to a previous prospective longitudinal study where 30% of people exhibited at least mild depression symptoms at both the baseline and follow-up [19]. Another qualitative descriptive study revealed that, in addition to psychological distress, caregivers also face other difficulties, such as sleep deprivation [20].

The prevalence of poor sleep quality for stroke patients in this study was 65%. This finding was lower compared to the results of previous studies. A cross-sectional study found that the prevalence of poor sleep quality was 84% (95% CI: 75–91%) [13]. Another cross-sectional study reported that a total of 25% of patients had sleep difficulties [10]. The explanation for this discrepancy in the sleep-quality score in our sample might be related to the duration since stroke onset, as the average time since stroke onset was nearly two years. Studies show that most stroke patients start to regain strength and cope with their new life conditions after the acute stage [10].

The prevalence of poor sleep quality among caregivers in this study was 46.3%. This finding was higher compared to a previous cross-sectional study, with results of 31% [21]. Moreover, another study confirmed that there was a decline in the duration, level of satisfaction, and quality of sleep for caregivers of stroke survivors [22]. The sleep-quality score in our study is higher than previous studies and was moderately associated with patient scores suggesting poor sleep quality. The selection of a convenience sample of caregivers who were willing to participate in this study might explain this discrepancy.

This study also found a strong correlation between PSDS and poor sleep quality in the patient sample (*r* = 0.885, *p* < 0.001). Our result is consistent with those of a previous study, which reported that sleep quality and depressive symptoms, as well as functional status, were considerably correlated [30]. Several studies reported that depression could result in poor stroke outcomes and delay survivors’ recovery.

As for caregivers, the study findings demonstrated a strong association between depressive symptoms and poor sleep quality (*r* = 0.669, *p* < 0.001). Furthermore, these results are supported by a previous study that reported that poor sleep and psychological distress in caregivers were significantly positively correlated (*r* = 0.619, *p* < 0.001) [21]. Caregivers provide emotional and social support to stroke survivors. However, sudden changes in stroke survivors’ medical conditions result in a burden for caregivers.

In fact, the disparity in the prevalence of depressive symptoms and sleep quality depended on the setting in which participants were enrolled, research design, data collection methods, duration since stroke onset, time of data collection, and instruments used.

### 4.1. Limitation of the Study

This study had some limitations that warrant discussion. For example, a cross-sectional design and a convenience sample were used. The data were collected over the phone using a survey, which led to some missing data. Participants who accessed the survey link and agreed to participate but did not complete the survey were excluded from the study. All participants included in the study had no missing data. Furthermore, insomnia is a symptom of depression as it is one of the assessment criteria for depression in the PHQ-9, therefore future consideration is needed to account for the collinearity between these constructs. In our study, we performed Pearson correlation without controlling for covariance. Therefore, further statistical analysis such as ANOVA or linear regression is highly recommended. In this study, we also excluded patients with cognitive impairments and aphasia. However, this study has several strengths, such as the use of an adequate sample of 100 stroke survivors and 80 caregivers in which the recommended sample size was 80 participants with a power of 80% and α error of 0.05. Data were collected by four researchers, and multiple correlation analyses between stroke survivors and the caregiver sample were conducted.

### 4.2. Implications and Recommendations for Nursing Practice

The findings of this study shed light on post-stroke complications among stroke survivors and caregivers in Saudi Arabia. The findings of this study will assist nurses in focusing on early screening for PSDS and the identification of patients and caregivers with poor sleep quality. Regular follow-up and a home healthcare plan are needed to enhance post-stroke health outcomes. Moreover, this study’s findings will help to increase awareness through educational programs about PSDS and find ways to enhance sleep quality. In addition, the outcomes of this study will help future researchers to use other methods of data collection and different tools and include more settings. The results will also be useful for future interventional studies aiming to improve sleep quality and prevent depression.

## 5. Conclusions

Poor sleep quality and depressive symptoms were found to be common among stroke survivors and their caregivers. This study contributes further information regarding the strong positive association between depressive symptoms and poor sleep quality among stroke survivors and their caregivers. Therefore, the identification of depressive symptoms and sleep disturbances in stroke patients and their caregivers should receive more attention. Future research should focus on an interventional study to assess the effectiveness of several methods to enhance sleep quality and decrease the risk of depressive symptoms.

## Figures and Tables

**Table 1:** Demographic characteristics of the sample.

Characteristics		Patients N = 100 N (%)	Caregivers N = 80 N (%)
Sex	Male	59 (59)	35 (43.8)
	Female	41 (41)	45 (46.3)
Nationality	Saudi	89 (89)	69 (86.3)
	Non-Saudi	11 (11)	11(13.8)
Marital Status	Married	66 (66)	49 (61.3)
	Single	11 (11)	25 (31.3)
	Widowed	13 (13)	3 (3.8)
	Divorced	10 (10)	3 (3.8)
Education	Elementary school	16 (16)	2 (2.5)
	Middle school	17 (17)	3 (3.8)
	High school	35 (35)	20 (25)
	University	16 (16)	46 (57.5)
	Higher degree	3 (3)	5 (6.3)
	Other	13 (13)	4 (5)
Employment	Employed	26 (26)	46 (57.5)
	Unemployed	16 (16)	10 (12.5)
	Retired	26 (26)	10 (12.5)
	Housewife	32 (32)	14 (17.5)
Monthly salary (Saudi riyal)	<2000	19 (19)	5 (6.3)
	2000–5000	17 (17)	19 (23.8)
	5000–10,000	14 (14)	22 (27.5)
	>10,000	13 (13)	18 (22.5)
	Do not know	37 (37)	16 (20)
City	Jeddah	92 (92)	72 (90)
	Makkah	3 (3)	-
	Other	5 (5)	8 (10)
Relation to patient	Father	-	27 (33.8)
	Mother	-	29 (36.3)
	Spouse	-	12 (15)
	Sibling	-	7 (8.8)
	Other	-	5 (6.3)
Hospital	KFGH	55 (55)	-
	KAMC	45 (45)	-
Stroke duration	<Year	30 (30)	-
	One year	26 (26)	-
	>Year	44 (44)	-

Note: KFGH = King Fahad General Hospital; KAMC = King Abdullah Medical Complex.

**Table 2:** Medical characteristics of the sample.

Characteristics	Patients N = 100 N (%)	Caregivers N = 80 N (%)
Diabetes Mellitus	60 (60)	13 (16.3)
Hypertension	65 (65)	20 (25)
Heart disease	16 (16)	7 (8.8)
Kidney disease	6 (6)	1 (1.3)
Hyperlipidemia	35 (35)	9 (11.3)
Obesity	8 (8)	2 (2.5)
Smoking	16 (16)	15 (18.8)
History of stroke	18 (18)	-
Other	7 (7)	4 (5)

**Table 3:** PHQ-9 score for stroke survivors and caregivers.

		Patient N (%)	Caregiver N (%)
PHQ-9 severity	0–4 None	27 (27)	29 (36.3)
	5–9 Mild	23 (23)	28 (35)
	10–14 Moderate	30 (30)	13 (16.3)
	15–19 Moderately Severe	14 (14)	10 (12.5)
	20–27 Severe	6 (6)	0

**Table 4:** Sleep quality score (PSQI) and PHQ-9.

	Patient				Caregiver			
	Minimum	Maximum	Mean	*SD*	Minimum	Maximum	Mean	*SD*
PSQI	0	17	7.85	3.622	0	14	6.16	3.046
PHQ-9	0	23	9.56	5.823	0	19	6.96	4.753

Note: PSQI = Pittsburgh Sleep Quality Index; (PHQ-9) = Patient Health Questionnaire.

**Table 5:** Prevalence of poor sleep quality and depressive symptoms.

	Score	Patient N (%)	Caregivers N (%)
PHQ-9	≥10	43 (43)	17 (21.3)
	<10	57 (57)	63 (78.8)
PQSI	>5	65 (65)	37 (46.3)
	<5	35 (35)	42 (52.5)

Note: PSQI = Pittsburgh Sleep Quality Index; (PHQ-9) = Patient Health Questionnaire.

**Table 6:** Pearson correlation of PSQI and PHQ-9 for patients and caregivers.

	1	2	3	4
1—PHQ-9 for patient	-			
2—PSQI for patient	0.885 **	-		
3—PHQ-9 for caregivers	0.502 **	0.492 **	-	
4—PSQI for caregivers	0.456 **	0.407 **	0.669 **	-

Note: ** Correlation is significant at the 0.001 level (one-tailed).

## Data Availability

The data used to support the findings of this study are available from the corresponding author.
